# Correlations between in vivo and in vitro tests with commercial extracts and fresh foods and specific IgE, in children with food allergy

**DOI:** 10.1186/2045-7022-1-S1-P96

**Published:** 2011-08-12

**Authors:** Mirjana Zivanovic, Marina Atanaskovic-Markovic

**Affiliations:** 1 Special Hospital Sokobanja, Allergology, Sokobanja, Serbia; 2 University Children's Hospital od Belgrade, Pediatric-Alergollogy, Belgrad, Serbia

## Background

The incidence of food allegy in children seems to be approximantely 6 to 8% in developed countries.The diagnosis of food allergy has to be confirmed by skin test, by performing specific IgE and by food challange.

## Aim

The aim of this study was to assess the correlations between results obtained with skin prick tests (SPT) using commercial extracts and prick-prick test (PPT) with fresh food, and the correlations between these results and those obtained with specific IgE.

## Methods

We performed a retrospective review of 249 children reffered to the University Children's Hospital of Belgrade for assessment of food allergy (cow's milk, hen eggs, wheat, peanuts, soybeans and kiwi) between 2008 and 2010. Children underwent cutaneous (SPT, PPT), serologic (Specific IgE) diagnostic and provocative test with commercially aviable allergen reagens and extracts.

## Results

132 (53%) SPT were assessed as being positive: 33 (47.8%) for CMP, 29 (51.7%) for egg white, 25 (44.6%) for egg yolk, 21 (47.7%) for peanuts, 11 (39.2%) for wheat, 9 (33.4%) for soybeans, 4 (16%) for kiwi. 211(85%) PPT were assessed as being positive: 50 (72.5%) for CMP, 41 (73.2%) for egg white, 37(66.07%) for egg yolk, 27 (61.4%) for peanuts, 21 (75%) for wheat, 16 (59.25%) for soybeans, 19 (76%) for kiwi. Specific IgE levels were being pozitive in 228 (91.5%) children. The conformable between a positive SPT and serum measurement specific IgE was 57.8% and the conformable between positive PPT and serum measurement specific IgE was 92.5%.

## Conclusion

Fresh food extracts are more effective in detecting sensitization. We obtained better conformable between fresh food tests and specific IgE, than with commercial extracts and measurement specific IgE.

